# On the Willingness to Pay for social media/messenger services taking into account personality and sent/received messages among WhatsApp users

**DOI:** 10.1016/j.heliyon.2024.e28840

**Published:** 2024-03-31

**Authors:** Christopher Kannen, Cornelia Sindermann, Christian Montag

**Affiliations:** a Department of Molecular Psychology, Institute of Psychology and Education, Ulm University, Ulm, Germany; b Computational Digital Psychology, Interchange Forum for Reflecting on Intelligent Systems, University of Stuttgart, Stuttgart, Germany

**Keywords:** Data business model, Surveillance capitalism, Personality, Alternative payment models, WhatsApp, Messenger services, Social media

## Abstract

WhatsApp has billions of users worldwide. Instead of paying a subscription fee, users provide their data for the use allowance. This data is used by Meta – the company behind WhatsApp – to obtain insights into user characteristics and monetize those insights. However, this data business model is among others criticized for fostering a loss of privacy that arises when platforms analyze user data, and for the use of design elements to attract users to the platform when they are not online or to extend their online time. Therefore, an increasing number of scientists are discussing whether other payment models are needed to overcome those disadvantages, like a monetary payment model. However, users would probably only pay for improved social media products. This paper provides an empirical basis for understanding the user perspective and, in particular, whether and how much users are willing to pay for improved social media products. For this, 2924 WhatsApp users’ perspectives on this topic were investigated. They were asked whether and how much they are willing to pay money for a messenger/social media service when its quality would be improved. Variables potentially influencing Willingness to Pay (i.e., personality, sent/received messages) were studied as well.

47% of the participants were unwilling to pay for a healthier messenger service, and about a quarter were willing or stayed neutral. Further analysis revealed that more agreeable people were more willing to pay. Further: Higher Extraversion was associated with more sent/received messages, but the number of sent/received messages was not linked to Willingness to Pay. The present study shows that many users still are not willing to pay for social media (here messengers), which indicates that the advantages of paying for social media with money instead of with one's own data might need to be better communicated.

## 1 Introduction

Over 4.7 billion active social media users have been counted as of July 2022, with a growth rate of 5.1% per year [1]. While years ago social media was considered an online website where users create profiles of themselves and stay in touch with friends, in the meanwhile platforms have developed in more diverse directions. The relevant features and activities of current social media platforms include messaging functions, video watching, curation of online profiles, and content sharing. The lines between different online services are increasingly blurred, and providers combine many features into a single application. Social media has also moved from the classical world wide web and internet browsers to smartphones in the form of applications and is even finding its way into virtual reality [40]. Against this background, it is not easy to define social media, but a prominent definition suggests that social media represents platforms where users can present themselves either in real time or asynchronously and speak from one to a few or many [2]. Also, messengers with functionalities like read receipts or message histories can be considered as belonging to the social media category [3].

### 1.1 WhatsApp

With over 2 billion users, WhatsApp is probably the most popular example of a messaging app [4]. It was founded in 2009 and was taken over by Meta (then Facebook) in 2014. The large userbase makes WhatsApp a highly visible and successful social media tool [4]. While some might see WhatsApp simply as a messenger but not a social media tool, given the opportunities to update one's own status and to speak from one to many synchronously or asynchronously, messenger applications such as WhatsApp can be considered social media products (again, see the social media definition [2]).

### 1.2 Data business model

The prevailing business model behind many mainstream social media platforms like WhatsApp foresees that users do not have to pay money for the allowance to use such a service. Instead, platform operators monetize their services with a data business model. Under this model, users provide their data in return for a use allowance, including data about the people with whom they are connected on the platform, what they are reading or posting, time of status updates, and so-called Likes provided to different topics, to name a few [41]. Social media companies analyze such data and, based on the emerging insights, display personalized advertisements to users. This procedure is known as micro-targeting [5]. The marketing industry pays money for these ad-opportunities to social media companies because micro-targeting seems to be more effective than rolling out the same ads to all users [6,7].

Data business models are highly profitable for companies. The market for advertisements on digital devices amounted to 522.5 billion USD in 2021 and is expected to grow by 60% by 2026 [8].

### 1.3 Risks of data business models

Despite the attractiveness of the mentioned data business model for social media companies, it has been proposed that this model comes with great costs for users and societies around the globe. Shoshana Zuboff mentioned that we live in the age of surveillance capitalism, where companies like those behind social media platforms spy upon their users as part of their business model [9,10]. Additional costs arising from the data business model include the prolongation of online time of users [11]. More and more researchers publish study results showing that excessive use of social media correlates with poor emotional health [12] and in this realm terms like Social Network Use Disorder (SNUD) are discussed [13]. Methods deployed by the industry to reduce online time, such as time keeper features, can be seen critical as narratives to move the responsibility for the users’ well-being from the industry to only the users themselves [11]. Abstaining from the data business model seems to be a crucial intervention to support humans in spending less time on social media [14]. Excessive usage not only includes the risk of developing addictive-like use tendencies: When companies generate and display content, that is highly interesting for users, this might also lead to prolonging proliferation of misinformation campaigns [15].

Against this background, it is increasingly questioned whether it is time to re-start social media, hence, to think about establishing healthier and improved social media platforms with the aim of reducing the negative side effects of the current data business model [16]. To find a solution, the data-business model must likely be abandoned. In a recent study based on interviews, experts pointed out that they consider alternative payment methods for social media, such as subscription-fees or public service social media, as a solution to tackle the aforementioned problems [17]. Without a change to alternative payment models, social media companies are likely to continue trying to seek data, prolong active online time, and obtain as much insight as possible into user characteristics accordingly. Although, as described above, the responsibility for healthy social media usage should not be transferred to the users (“victim blaming” [14]), of course the users’ perspective on this topic is very important to find out if users see this topic as problematic as well.

### 1.4 Context of the present study

Recent studies wanted to understand the view of users on the data business model and alternatives [16,18]. They investigated how many participants of their study would be willing to pay for a social media service when platforms would operate differently and respect the rights of their users and how much money per month they would be willing to pay [19]. In this recent study, it was observed that 49.52% of the participants would not be willing to pay money for an alternative social media service, and 21.43% of the study participants opted to pay with money, and 29.05% stayed neutral. This initial work also observed that the personality trait of Agreeableness, hence, being more cooperative and empathic, was associated with a higher Willingness to Pay with money for a social media service. As the work by Sindermann et al. [19] was based on only few participants (>200), the authors deemed their results to be preliminary. Because those results were preliminary, the aim of the present work was to revisit the earlier findings of Sindermann et al. [19] in a much larger sample.

For that, the question about Willingness to Pay was put in the context of users of a prominent product that is representative of many social media services used daily: WhatsApp [20]. The present study was willingly rolled out to attract WhatsApp users, because this platform represents the most prominent messenger platform worldwide – at the moment counting more than two billion users (see above). In addition, the aim of the study was to investigate the relationship between Willingness to Pay for social media/messenger services and intensity of WhatsApp use as an extension of the previous study.

### 1.5 Hypotheses and exploratory analyses

As one can derive from the Agreeableness-Willingness to Pay associations found in Sindermann et al. [19], personality might be relevant in this context and therefore, the personality-complex is studied again in the present work. Personality refers to rather time-stable characteristics of a person manifesting in cognitive, emotional, motivational, and behavioral tendencies [21], which is reflected also on in this work – by studying the well-known Big Five Model comprising the broad personality dimensions Neuroticism, Extraversion, Openness to Experiences, Agreeableness and Conscientiousness (stemming from a lexical approach [22]).

While a positive relation between Agreeableness and Willingness to Pay can be hypothesized based on Sindermann et al. [19], the other Big Five relations are investigated exploratively.

Going beyond the study by Sindermann et al. [19], we also investigate WhatsApp activity by considering the number of messages received and sent via the messenger. It is likely that users who are more active on WhatsApp (in terms of more received/sent messages) are more willing to pay for a social media/messenger product.

Since we also collected personality data, the present work provides a unique opportunity to explore how personality is linked to WhatsApp activity in a study with a large sample size. Based on previous research we hypothesized that Extraversion is linked to more WhatsApp usage [23,24]. We expected the number of received/sent messages on WhatsApp to be positively related to Extraversion. Since the data collection ran over three years, we also looked whether the answer behavior regards Willingness to Pay (WtP) changed over time, especially against the background of the pandemic.

## 2 Methods and materials

The present work is part of a larger project, consisting of a cross-sectional study, whose results are (in parts) presented in this paper, and a longitudinal study with an experiment, where users were asked to change their WhatsApp usage for one week to observe changes in certain measures (the longitudinal part of the study has been preregistered: https://osf.io/pdvq5). Therefore, further papers from this large project will be published in the near future. The overarching aim of the scientific project aimed to understand WhatsApp use with a focus on the data business model and a possible link between design elements such as read receipt functions and well-being (the latter not being focus of the present work). A small set of the personality data has been published in an earlier work dealing not with a social media topic [42].

The investigation of links between personality and sent/received messages can be seen as part of the digital phenotyping movement [25,26] which is closely related to the term digital computing [27]. In this movement, researchers among others aim to predict psychological characteristics from digital footprints (here the number of messages received and sent).

### 2.1 Participants

*N* = 3374 participants were initially recruited for the present online study from May 2020 to January 2023. The study was promoted via different online channels, including news websites and social media, as well TV and radio appearances of the authors. The study was framed as a WhatsApp study and addressed only WhatsApp users. Participants that did not use WhatsApp were either directly excluded from the study by some control questions or manually removed during the data cleaning (see below) if their answers indicated, that they are no WhatsApp users. Participants were not paid for participation. Instead, they were incentivized with feedback about their own results from the survey compared to the anonymous and averaged answers of the other participants. This feedback was shown to each participant after completing the study. Prior to the survey, all participants provided informed e-consent and needed to declare to be 13 years of age or older. The study protocol asked participants of minor age to discuss the present study with their parents before participation. And only if both, parents and study participants, agreed upon participations (indicated by a checkbox) participation was possible. Due to the anonymous online character of the study no paper consent could be collected. This study was approved by the local ethics committee of Ulm University (93/20).

### 2.2 Data cleaning

The first 77 participants completed a test version of the survey and were therefore excluded from the final analyses. Further, eight participants were excluded because they were younger than 13 years (the minimum age for participation), and one was excluded, providing a non-meaningful age of 2000 years. Although this was likely the year of birth, we decided to remove this individual from the final dataset to avoid making assumptions. As the present study stems from a larger research project with a focus on WhatsApp usage (on smartphones), only people that reported to have a WhatsApp account were enrolled. To check this, we asked multiple times if and how often participants used WhatsApp or messengers in general. *n* = 186 participants who reported having WhatsApp not installed on their phones, not using it, or not using any messenger apps were excluded. As mentioned in the Introduction, the participants also provided information about the number of messages they had sent or received on WhatsApp. We provided detailed instructions on how exact numbers could be read from WhatsApp statistics. Three participants were excluded because they entered “0” or left the field blank. We also found that some people guessed the numbers because they entered extremely high numbers (even millions) as count of WhatsApp messages. We decided to calculate a WhatsApp messages per month variable and excluded participants who sent more than 30,000 messages per month or received more than 90,000 messages per month. Those numbers seem appropriate as upper limits, since they correspond to 1000 written messages per day or approximately one written message per minute, assuming 8 h of sleep and 16 h being awake. For the received messages, we calculated with 3× the number of messages, since it is very likely that more messages are received than are sent due to the feature of WhatsApp groups.

This filter led to another 78 participants that were removed. Finally, 97 participants were removed because they answered questions about how many years or months, they already used WhatsApp or had it installed with a time span starting before the existence of WhatsApp (>12 years for those who have completed the survey in 2020, >13 years for 2021, and so on). As for the one person removed because of his/her age (2000 years), it seemed clear for many of those participants that they also answered with the year when they started to use WhatsApp. However, we removed them for the same reasons. Nobody was excluded due to careless responses (choosing the same answer option for all items presented on one page of the Big Five inventory; hence across a span of 45 items here) or missing values. Please note that participants were asked to respond to all items: If they did not respond to all items at first, the survey software asked them automatically to fill in the missing items, otherwise they could not proceed to the next page of the survey. If a participant did not want to respond to all items, the participant could terminate participation at any time without any consequences.

### 2.3 Final dataset

After the mentioned steps of data cleaning, around 15 % of the participants were removed and the final sample size was 2924 (*n* = 1136 males, *n* = 1788 females). The mean age was 24.36 years (*SD* = 7.79, median = 23.00). Most participants (*n* = 1,174, 40.2%) answered that they had a high school degree (German: “Abitur”) as the highest educational level. A total of 929 (31.8%) participants had some kind of university degree and the remaining participants (*n* = 821, 28.1%) had a lower or no educational degree. Percentages do not add up to 100% due to rounding inaccuracies. The sample included many young participants who had not graduated from school at the time of completing the survey and, for that reason, had no educational degree.

### 2.4 Questionnaires and materials

All the participants completed several questionnaires as part of a larger project. The relevant questionnaires for the present work are presented below.

#### 2.4.1 Willingness to Pay for social media (WtP)

We included a four-item questionnaire to assess whether participants were willing to pay a monthly monetary fee for any kind of social media if thereby certain aspects of the platform were improved. The four different improvements presented in separate items were: i) the user's personal data will not be used for marketing, ii) the user's personal data will be better protected, iii) the platform providers will not design their applications in a way that encourages longer online time, and iv) fake news and radicalization will be reduced on the platform. Possible answer options ranged from 1 (strongly disagree) to 5 (strongly agree) on a five-point Likert scale. All four questions were bundled into one variable (WtP) by taking the average of the answer values (Cronbach's alpha = 0.87, McDonald's omega = 0.91). Scores below or equal to 2.5 points were treated as indicating no or little Willingness to Pay, and scores equal to or above 3.5 points as a Willingness to Pay. Scores in between were treated as indicating a neutral stance. This procedure is almost in line with the original work by Sindermann et al. [19], where the groups were set to: 1.00–2.50: not willing to pay; 2.51–3.50: neutral; 3.51–5.00: willing to pay. Reasons for the slightly adjusted grouping and the comparison of the different calculation methods can be found in the Supplementary Material.

#### 2.4.2 Willingness to Pay amounts for messengers (WtPA-MSG) and social media (WtPA-SM)

Two more items were administered asking participants how much money they were willing to pay for a social media platform in broad and, more precisely, for a messenger service per month to increase the quality, as defined in the WtP section. As discussed earlier, social media definitions exist that categorize WhatsApp as belonging to the umbrella term social media [2]. As this is debatable, at least in this part of the study, we asked participants about the exact money they would be willing to pay for social media and messengers with separate questions. Because this item was an open-ended question, some individuals inserted very high numbers. We winsorized outliers according to the formula by Tukey [28] [Smaller: 25% Quantile − 1.5 × (75% Quantile − 25% Quantile); Greater: 75% Quantile + 1.5 × (75% Quantile − 25% Quantile)] and therefore exchanged outliers for 36 participants (1.2%) in the upper range, with a cut-off value (10€) for the WtPA-MSG variable. There were no outliers for smaller values. For WtPA-SM, 113 answers (3.9%) were winsorized to a value of 7.50€, which is the cut-off value for this item with regard to the Tukey formula.

#### 2.4.3 BFI-45 to assess the Big Five of personality

The Big Five were assessed using the BFI-45 (German language [29]). In line with the international version, we used only 44 items for better comparability. The questionnaire assesses with 10 items, the personality dimension Openness to Experience (Cronbach's alpha = 0.80, McDonald's omega = 0.85) with 9 items, the personality dimension Conscientiousness (Cronbach's alpha = 0.83, McDonald's omega = 0.87) with 8 items, the personality dimension Extraversion (Cronbach's alpha = 0.86, McDonald's omega = 0.89) with 9 items, the personality dimension Agreeableness (Cronbach's alpha = 0.71, McDonald's omega = 0.77), and finally, the personality dimension Neuroticism (Cronbach's alpha = 0.84, McDonald's omega = 0.88) with 8 items. The questionnaire was administered using a five-point Likert scale ranging from 1 (disagree strongly) to 5 (agree strongly). Higher scores indicate higher personality traits in each dimension (after recoding negatively formulated items and calculating the mean value for each dimension).

#### 2.4.4 Activity on WhatsApp (WhatsApp-SENT/WhatsApp-RECV)

The WhatsApp activity was assessed by asking how many messages each participant had sent or received via their accounts. These statistics were not estimated by participants, but each participant looked up the individual numbers on WhatsApp messages in the WhatsApp settings. We provided instructions on how these numbers could be found. Because such statistics can be set back, we also asked if this was the case (yes = 1, no = 2) and if yes, how many months ago this happened. In addition, we asked how many months the app had been installed on the present smartphone and how long WhatsApp had been used in general (in years). With the combination of these answers, we were able to calculate the average number of WhatsApp messages sent per month (WhatsApp-SENT) and WhatsApp messages received per month (WhatsApp-RECV). This was done by taking the count of each sent and received message and dividing them by the smallest number of the time variables per user (either the re-installation time or reset time). The result of this calculation is the average monthly number of messages sent or received from the current phone since WhatsApp was installed on that phone. Since the values were also widely spread, we decided to winsorize them according to the formula of Tukey [28], as described above. A total of 323 (11.0%) sent WhatsApp message answers were winsorized to the cut-off value of 5091.1 messages per month and 346 (11.8%) of the received WhatsApp message answers were winsorized to the cut-off value of 9219.5 messages per month.

### 2.5 Statistical analyses

First, descriptive statistics were computed. In this process, the suitability of parametric or non-parametric testing was checked. The variables WtPA-MSG, WtPA-SM, WhatsApp-SENT, and WhatsApp-RECV showed skewness greater than ± 1 and or kurtosis greater than ± 3, which indicates a non-normal distribution of the data. Accordingly, non-parametric tests were applied whenever these variables were investigated [30]. The same was true for the age distribution. In addition, non-parametric tests were used when education was included because of the ordinal answer scale. For the BFI-45 and WtP questionnaires, the answer options are on a Likert scale and, hence, ordinal. However, the computed BFI factors and combined WtP scale scores were interpreted as interval-level measurements. Parametric tests were applied to these variables, wherever possible.

Correlations were computed using Pearson's correlation coefficient to find associations between personality traits and WtP. Further, correlations between the Big Five and the single WtP questions were measured with Spearman's correlation, just as the correlation between WtP and the date of participation (in the form of months relative to the study start in May 2020). Date of participation was analyzed because the data collection happened while the COVID-pandemic was going on and this was done to see if time of the pandemic somehow influenced our findings regarding the central variable. Spearman's correlation coefficients were used to investigate the correlations between personality factors and the four variables WtPA-MSG, WtPA-SM, WhatsApp-RECV, and WhatsApp-SENT. Spearman's method was also applied for testing correlations between WtPA variables, WhatsApp usage variables, age and education. The *t*-test was used to test the relation of sex and WtP. The necessity to apply the Welch t-test was tested, but it was not necessary. Mann-Whitney-U tests were computed to test the relation of sex and the two WtPA variables and the two WhatsApp usage variables. Finally, regression models were computed to investigate the relations with WtP using the following approach: For each of the predicted variables, three regression models were set up with different predicator variables: Model 1 – Demography (Age, Sex, Education); Model 2 – Sent and received WhatsApp messages (WhatsApp-SENT and WhatsApp-RECV); Model 3 – the Big Five scales. In a fourth model, all predictor variables from the first three regression models were included (Age, Sex, Education, WhatsApp-SENT and WhatsApp-RECV, Big Five scales) into one. For all regression models, WtP, age, sent and received messages and personality variables were z standardized. For the two regression models where Education was used as variable, it was grouped into two groups (1. No degree or any kind of school degree; 2. Any kind of university degree).

All analyses were also computed separately for males and females to investigate sex differences. As a general rule of thumb, we report correlations equal to or higher than 0.10 in the text. In rare cases we deviate from this approach, when it sheds further light on relevant research questions. All other results can be found in the Supplementary Material.

### 2.6 Software

All computations were run on R (version 4.2.3) with the following packages: dplyr (version 1.1.1), expss (version 0.11.4), fmsb (version 0.7.5), foreign (version 0.8.84), ggplot2 (version 3.4.1), GPArotation (version 2023.3.1), gridExtra (version 2.3), haven (version 2.5.2), labelled (version 2.10.0), lsr (version 0.5.2), ltm (version 1.2.0), lubridate (version 1.9.2), moments (version 0.14.1), psych (version 2.3.3), sjPlot (version 2.8.13). Fig. 2 was created with Jamovi (version 2.3.21.0) with the following library installed: jjstatsplot (version 0.0.2).

## 3 Results

### 3.1 Descriptive statistics and differences between males and females

The descriptive statistics for all relevant variables are presented in Table 1. As one can see, in this sample, females reported on average to be more open, more conscientious, more extraverted, more agreeable, and more neuroticistic than males. Furthermore, females sent more messages and received more messages via WhatsApp. Finally, they were more willing to pay for social media than males and were also willing to pay a higher subscription fee for social media (not messengers) than males. This supports the idea of presenting the dataset not only on total sample levels but also on the level of male and female subsamples.

**Table 1:** Descriptive statistics including sex differences.

	Total Sample (*N* = 2924)		Males (*n* = 1136)	Females (*n* = 1788)	Sex Differences
	*M* (*SD*)	Median	*M* (*SD*)	*M* (*SD*)	
**Openness**	3.55 (0.65)	3.60	3.49 (0.66)	3.59 (0.65)	*t* = −3.76, *p* < 0.001, *d* = 0.14
**Conscientiousness**	3.39 (0.69)	3.44	3.29 (0.68)	3.46 (0.68)	*t* = −6.79, *p* < 0.001, *d* = 0.26
**Extraversion**	3.23 (0.80)	3.25	3.14 (0.81)	3.29 (0.79)	*t* = −4.84, *p* < 0.001, *d* = 0.18
**Agreeableness**	3.52 (0.57)	3.56	3.40 (0.57)	3.59 (0.56)	*t* = −8.92, *p* < 0.001, *d* = 0.34
**Neuroticism**	3.05 (0.79)	3.00	2.81 (0.78)	3.20 (0.75)	*t* = −13.50, *p* < 0.001, *d* = 0.51
**WtP**	2.72 (1.12)	2.75	2.65 (1.17)	2.76 (1.07)	*t* = −2.52, *p* = 0.012, *d* = 0.10
**WtPA-MSG**	2.43 (2.60)	2.00	2.30 (2.56)	2.51 (2.62)	*W* = 4,181,524.00, *p* = 0.132, *r* = −0.03
**WtPA-SM**	1.66 (2.12)	1.00	1.58 (2.11)	1.71 (2.12)	*W* = 5,318,030.00, *p* < 0.001, *r* = −0.31
**WhatsApp-SENT**	1465.34 (1685.87)	725.43	1372.01 (1689.84)	1524.64 (1681.13)	*W* = 92,284.00, *p* < 0.001, *r* = −Inf
**WhatsApp-RECV**	2743.05 (3055.74)	1416.28	2730.09 (3100.51)	2751.29 (3027.79)	*W* = 61,740.00. *p* < 0.001, *r* = −Inf

**Table abbreviations**.

**WtP:** Willingness to Pay (Average value calculated from 4 questions with Likert-type answer options).

**WtPA-MSG:** Amount per month the participant offered to pay for a single messenger platform (free text field for answer).

**WtPA-SM:** Amount per month the participant offered to pay for a single social media platform (free text field for answer).

**WhatsApp-SENT:** Sent WhatsApp messages per month, calculated from the total sent WhatsApp messages and the time since when WhatsApp is used on the specific device (variable was winsorized).

**WhatsApp-RECV:** Received WhatsApp messages per month, calculated from the total received WhatsApp messages and the time since when WhatsApp is used on the specific device (variable was winsorized).

### 3.2 More detailed descriptive statistics for WtP

**Fig. 1:**
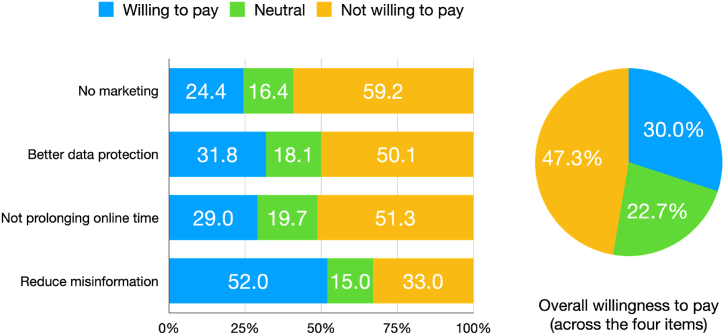
Willingness to Pay for a social media service on item level (left side) and averaged across items (right side).

As illustrated in the pie chart of Fig. 1, many of the participants *n* = 1383 (47.3%) were counted for the group of people that are not willing to pay for social media (average WtP score across four items ≤ 2.5). 664 (22.7%) answered neutrally on average (2.5 < WtP < 3.5), and 877 (30.0%) answered that they were willing to pay for social media services (WtP≥3.5).

**Fig. 2:**
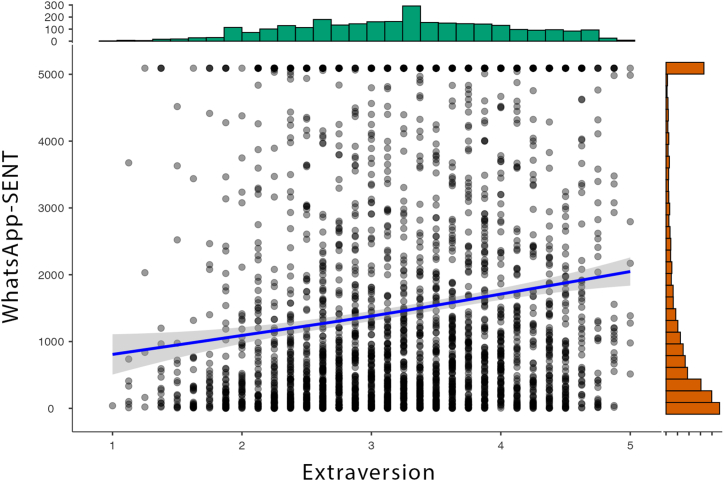
Distribution of Sent WhatsApp Messages (top) and Extraversion (right side) and the scatterplot showing associations between both variables (dot chart).

Of all participants, 421 (14.4 %) answered that they were strongly against paid services for all four questions (WtP score = 1, because all questions were answered 1 = strongly disagree).

90 participants (3.1%) answered strictly with the fifth option (WtP score = 5, because all questions were answered with 5 = strongly agree) and, hence, strongly agreed to pay for social media for all four reasons.

When looking at the answers to the four Willingness to Pay questions individually, the results ranged from 24.4% to 52.0% (see left side of Fig. 1) participants choosing response option 4 or 5. These results show the highest Willingness to Pay (blue area, questions answered with answer option 4 or 5 in Fig. 1, left side) when misinformation campaigns are reduced and the lowest when marketing opportunities are reduced. The highest Unwillingness to Pay was found if data was not used for marketing purposes (yellow area, answer options 1 or 2); green area indicates neutral responses, answer option 3.

### 3.3 More detailed descriptive statistics of WtPA-MSG and WtPA-SM

For the two questions regarding how much a person is willing to pay, we see high peaks at 0€. *N* = 1330 (45.5%) entered 0€ for WtPA-SM (*M* = 1.66, *SD* = 2.12) and *n* = 813 (27.8%) entered 0€ for WtPA-MSG (*M* = 2.43, *SD* = 2.60). The maximum values after winsorization were 10€ for WtPA-MSG and 7.50€ for WtPA-SM.

### 3.4 Correlation patterns between WtP variables

Significant correlations were found between WtP and WtPA-MSG (*rs* = 0.382, *p* < 0.001), between WtP and WtPA-SM (*rs* = 0.559, *p* < 0.001) as well as between WtPA-MSG and WtPA-SM (*rs* = 0.516, *p* < 0.001).

### 3.5 Correlation patterns between WtP variables and WhatsApp messages

Correlations between the WtP score and sent/received messages were investigated but not significant. But both WtPA variables correlated with both WhatsApp message variables: WtPA-MSG correlated with WhatsApp-SENT (*rs* = 0.071, *p* < 0.001), WtPA-SM correlated with WhatsApp-SENT (*rs* = 0.074, *p* < 0.001), WtPA-MSG correlated with WhatsApp-RECV (*rs* = 0.062, *p* = 0.001), and WtPA-SM correlated with WhatsApp-RECV (*rs* = 0.073, *p* < 0.001).

### 3.6 Correlation patterns with age

For males, age was significantly correlated with WtPA-MSG (*rs* = −0.103, *p* = 0.001), and WtPA-SM (*rs* = −0.132, *p* < 0.001). Likewise, significant correlations, but smaller effect sizes, were found for the total sample between age and WtPA-MSG (*rs* = −0.083, *p* < 0.001) and WtPA-SM (*rs* = −0.084, *p* < 0.001) and for females with WTPA-MSG (*rs* = −0.067, *p* = 0.005).

Age was also significantly correlated with WhatsApp-RECV in the total sample (*rs* = −0.135, *p* < 0.001), and for males (*rs* = −0.136, *p* < 0.001) and females (*rs* = −0.134, *p* < 0.001).

Finally, age was significantly correlated with Agreeableness in the total sample (*rs* = 0.100, *p* < 0.001).

### 3.7 Correlation patterns with education

Education was significantly correlated with WtP (*rs* = 0.077, *p* < 0.001). The significant correlation was only found for females (*rs* = 0.112, *p* < 0.001), but not for males (*rs* = 0.018, *p* = 0.554). For males, education was also significantly correlated with WhatsApp-SENT, but effect sizes are very small (*rs* = 0.059, *p* = 0.048).

### 3.8 Correlation patterns with personality

As shown in Fig. 2, Extraversion correlated with WhatsApp-SENT (*rs* = 0.195, *p* < 0.001). Extraversion also correlated with WhatsApp-RECV (*rs* = 0.158, *p* < 0.001). Agreeableness correlated with WtP (*r* = 0.103, *p* < 0.001) and was also significantly related, but showed even smaller effect sizes, with WtPA-MSG (*rs* = 0.070, *p* < 0.001) and WtPA-SM (*rs* = 0.085, *p* < 0.001). Many other correlations with very small effect sizes <0.10 were observed and can be found in Table 2.

**Table 2:** Correlations between Big Five and several variables of interest (always calculated three times: 1. for the complete sample, 2. only for males, 3. only for females).

	Openness	Conscientiousness	Extraversion	Agreeableness	Neuroticism
**WtP**	*r* = 0.059, *p* = 0.001	*r* = 0.064, *p* < 0.001	*r* = 0.082, *p* < 0.001	*r* = 0.103, *p* < 0.001	*r* = −0.005, *p* = 0.806
**WtP (Males)**	*r* = 0.062, *p* = 0.038	*r* = 0.062, *p* = 0.038	*r* = 0.065, *p* = 0.029	*r* = 0.107, *p* < 0.001	*r* = 0.028, *p* = 0.344
**WtP (Females)**	*r* = 0.052, *p* = 0.029	*r* = 0.058, *p* = 0.015	*r* = 0.088, *p* < 0.001	*r* = 0.091, *p* < 0.001	*r* = −0.048, *p* = 0.041
**WtPA-MSG**	*rs* = 0.026, *p* = 0.155	*rs* = 0.021, *p* = 0.264	*rs* = 0.037, *p* = 0.046	*rs* = 0.070, *p* < 0.001	*rs* = 0.025, *p* = 0.182
**WtPA-MSG (Males)**	*rs* = 0.004, *p* = 0.881	*rs* = 0.023, *p* = 0.439	*rs* = 0.030, *p* = 0.319	*rs* = 0.060, *p* = 0.044	*rs* = 0.012, *p* = 0.689
**WtPA-MSG (Females)**	*rs* = 0.034, *p* = 0.150	*rs* = 0.010, *p* = 0.659	*rs* = 0.037, *p* = 0.115	*rs* = 0.060, *p* = 0.011	*rs* = 0.012, *p* = 0.621
**WtPA-SM**	*rs* = 0.029, *p* = 0.112	*rs* = −0.010, *p* = 0.578	*rs* = 0.046, *p* = 0.013	*rs* = 0.085, *p* < 0.001	*rs* = 0.052, *p* = 0.005
**WtPA-SM (Males)**	*rs* = 0.025, *p* = 0.399	*rs* = 0.005, *p* = 0.862	*rs* = 0.075, *p* = 0.012	*rs* = 0.081, *p* = 0.006	*rs* = 0.072, *p* = 0.015
**WtPA-SM (Females)**	*rs* = 0.029, *p* = 0.228	*rs* = −0.029, *p* = 0.218	*rs* = 0.023, *p* = 0.332	*rs* = 0.076, *p* = 0.001	*rs* = 0.029, *p* = 0.226
**WhatsApp-SENT**	*rs* = 0.005, *p* = 0.768	*rs* = −0.013, *p* = 0.481	*rs* = 0.195, *p* < 0.001	*rs* = −0.002, *p* = 0.902	*rs* = 0.083, *p* < 0.001
**WhatsApp-SENT (Males)**	*rs* = 0.055, *p* = 0.063	*rs* = −0.004, *p* = 0.883	*rs* = 0.231, *p* < 0.001	*rs* = −0.032, *p* = 0.281	*rs* = 0.021, *p* = 0.481
**WhatsApp-SENT (Females)**	*rs* = −0.034, *p* = 0.155	*rs* = −0.035, *p* = 0.135	*rs* = 0.166, *p* < 0.001	*rs* = −0.003, *p* = 0.902	*rs* = 0.097, *p* < 0.001
**WhatsApp-RECV**	*rs* = −0.006, *p* = 0.736	*rs* = −0.023, *p* = 0.214	*rs* = 0.158, *p* < 0.001	*rs* = −0.018, *p* = 0.332	*rs* = 0.063, *p* = 0.001
**WhatsApp-RECV (Males)**	*rs* = 0.029, *p* = 0.324	*rs* = −0.005, *p* = 0.861	*rs* = 0.186, *p* < 0.001	*rs* = −0.046, *p* = 0.119	*rs* = 0.008, *p* = 0.786
**WhatsApp-RECV (Females)**	*rs* = −0.032, *p* = 0.177	*rs* = −0.039, *p* = 0.097	*rs* = 0.138, *p* < 0.001	*rs* = −0.008, *p* = 0.724	*rs* = 0.094, *p* < 0.001

r = Pearson correlation, rs = Spearman correlation.

### 3.9 Correlation with month and year of participation

No significant correlations between the participation date and WtP were not observed.

### 3.10 Regression models to predict Willingness to Pay (WtP)

The first regression model, as depicted in Table 3 with demographic variables as predictors for WtP, showed a significant relation of sex with WtP, with low effect sizes and an adjusted r-squared value of 0.002. The second regression model with WhatsApp-SENT and WhatsApp-RECV as predictors of WtP did not show any significant relations of these predictor variables with WtP, while the third model based on personality traits showed significant relations of Agreeableness, Extraversion, Conscientiousness, and Neuroticism. The effect sizes were small, and the r-squared value was 0.018. In the fourth model, only the predicator variables Conscientiousness, Extraversion and Agreeableness showed significant relations with WtP and the r-squared value was 0.017

**Table 3:** Results of all four regression models built to predict Willingness to Pay (WtP) from demography, WhatsApp messages and personality traits.

DV	IVs	Estimate	*p*-value	F-statistic	DF	Multiple *R*-squared	Adjusted *R-*squared
**WtP (z)**	(Intercept)	−0.222	0.008				
	**Age (z)**	−0.001	0.971				
	**Sex**	0.093	0.014				
	**Education (2 groups)**	0.054	0.218				
			0.043	2.72	2920	0.003	0.002
							
**WtP (z)**	(Intercept)	0.000	1.000				
	**WhatsApp-SENT (z)**	0.053	0.230				
	**WhatsApp-RECV (z)**	−0.042	0.340				
			0.465	0.77	2921	0.001	−0.000
							
**WtP (z)**	(Intercept)	0.000	1.000				
	**Openness (z)**	0.031	0.103				
	**Conscientiousness (z)**	0.049	0.011				
	**Extraversion (z)**	0.065	<0.001				
	**Agreeableness (z)**	0.093	<0.001				
	**Neuroticism (z)**	0.040	0.040				
			<0.001	11.80	2918	0.020	0.018
							
**WtP (z)**	(Intercept)	−0.055	0.539				
	**Age (z)**	−0.006	0.757				
	**Sex**	0.015	0.708				
	**Education (2 groups)**	0.023	0.597				
	**WhatsApp-SENT (z)**	0.024	0.600				
	**WhatsApp-RECV (z)**	0.022	0.629				
	**Openness (z)**	0.031	0.110				
	**Conscientiousness (z)**	0.047	0.017				
	**Extraversion (z)**	0.063	0.002				
	**Agreeableness (z)**	0.091	<0.001				
	**Neuroticism (z)**	0.036	0.080				
			<0.001	5.96	2913	0.020	0.017

## 4 Discussion

The present study aimed to understand the users’ perspective on alternative business models for social media and messengers where users pay with a subscription fee instead of with their data.

In doing so, it investigated if preliminary findings showing that about 50% of study participants were unwilling to pay for a social media service, even if the service was improved by this (and about a quarter were neutral or willing to pay for such a service) could be revisited. These findings were investigated in a small sample of around 200 participants [19] before, and did require further investigation in a larger sample.

### 4.1 Willingness to Pay (WtP)

In the present work, about 47% of the participants were not willing to pay for a social media service if problems oftentimes associated with the current data business model would be reduced. This is about the same as observed by Sindermann et al. [19]. 30% were willing to pay (21% for the study by Sindermann et al.) and about 23% of the participants stayed neutral (29% for the study by Sindermann et al.) [19]. Please note that the numbers have been derived in the present work by a slightly different rule, therefore see also the supplement.

More fine-granular analysis revealed that the Willingness to Pay was also dependent on the problem area. Participants were more willing to pay for social media when misinformation campaigns were reduced (52.0%) compared to when marketing opportunities of the industry were reduced (24.4% were willing to pay for social media in this case).

Investigating the personality traits, the present study also showed that Openness, Conscientiousness, Extraversion and Agreeableness positively correlated with WtP. This replicates the results that more agreeable persons were generally more willing to pay for a social media service, but the effect sizes were around half the size of the initially observed ones in Sindermann et al. [19]. To more illustrate the effect size issue: Agreeableness showed the largest association with WtP and this was just around 0.10. Hence, this is about 1% shared variance between the constructs and a very small effect size.

Interestingly, users with more intense WhatsApp usage in terms of sent/received messages were not more willing to pay for social media.

The four regression models substantiated the findings regards WtP. Model 3, investigating the influence of personality on WtP, showed that personality traits can be used as predictors for WtP. However, the adjusted r-squared also shows that only 2% of the WtP variance can be explained with the personality traits (Agreeableness still showed the largest from the small personality associations with WtP; this was not only visible in the regression model, but also within the overarching correlation table with the presented bivariate associations). Demographic variables and the sent and received WhatsApp messages showed hardly any significant results and had a very low influence (not implying causality here) on WtP respectively no influence on WtP at all.

### 4.2 Willingness to Pay Amounts (WtPA)

Participants in this study were asked to enter the amount of money they were willing to pay per month to compensate a company for offering a single service with money instead of data.

The average amounts per month individuals were willing to pay in this study (2.43€ for messengers and 1.66€ for social media) were almost identical for messengers and 25% lower for social media compared to the previous study by Sindermann et al. [19].

Of further interest is our observation that the average amounts per service are not far-off the revenue that companies make with their business model nowadays. Meta for example published numbers for the Average Revenue per User (ARPU) for Facebook of USD 9.62 for Q3 2023 (around 2.92€ per month) [31] and for all Meta platforms combined of USD 39.63 for 2022 (around 36.00€ per year or 3.00€ per month) [32].

These figures show that the users' understanding of fair compensation is very realistic. Please also note that participants in this study were asked to enter amounts per service, with Meta's latter figures applying to all of Meta's products in combination. From this we can conclude that a change of business model could even be lucrative for social media companies. Users of multiple applications might pay for several services of one provider and thus increase their revenue.

### 4.3 WhatsApp usage

In addition to the age correlation with WtP amounts, there was also a negative correlation between age and WhatsApp usage. This indicates that messenger services are associated with more value for younger persons and that younger persons tend to use WhatsApp more frequently.

For scientists interested in predicting personality traits from digital footprints, it is interesting to note that higher Extraversion accompanied more sent/received messages. This fits also with observations showing that a) Extraversion can be in particular quite well inferred from digital footprints in the area of social media and smartphone data [24,33] and b) that not only social activities such as phone calls are associated with Extraversion [34], but also communicating via messenger services.

### 4.4 Alternative business models

The results of this study show that some users already are open to the alternative business model of paying with money instead of data. However, the overall prevalence of individuals being willing to pay is low.

It is also important to note that not every individual can or wants to afford a paid subscription for a social media account. This is in particular true considering that individuals oftentimes use more than one social media platform.

Considering all these numbers, a hybrid model might be a feasible alternative for both users and companies. In such a model, users could decide if they either want to continue providing extensive amounts of their data or pay with a subscription fee instead and keep more of their data private. When choosing the second option, the platform should also not aim at prolonging online time. But this would mean that the companies behind social media would need to redesign their platforms, to operate in parallel in different ways. At the moment it is questionable if such an offer will be made. Although this has been reported more often in the news recently: for instance, the German outlets “Die Zeit” or “Der Spiegel” offer such a choice to their users under the name of PUR-models. Further, Meta considers providing users in the EU (due to the newly introduced Digital Markets and Digital Services Act) the option to pay for ad-free Meta platforms [[35], [36]]. Such a model should, however, take into account that people on low incomes should not be pressured into disclosing their data. Further, it needs to be hindered that using a more healthy social media platform in the end is only accessible for privileged and rich users.

Hence, for any alternative model, it is also important to consider differences in sociodemographics including income level to not hinder users from onboarding social media. Therefore, the idea of social media as a public service, including price reductions for less-well earning individuals, should also be considered [17].

Independent from such a hybrid model, we believe that it is time to discuss general questions about social media, like if social media belongs in the hand of private companies at all, or if these platforms are so relevant for public discourse that they would be better off as public good [16].

### 4.5 Strength and limitations

The present work is based on an empirical study with a large sample size of *N* = 2924 participants. Therefore, sufficient statistical power was available to robustly carve out even small effects. Further, the present research focused on the study of users of one of the largest messenger platforms.

However, the present work also comes with several limitations. First, the study is of self-report nature and can come with issues such as tendencies to answer in a socially desirable fashion, and issues arising from the lack of introspection. Even the objective data are error prone when the participants failed to not transfer the correct statistics from WhatsApp to our online form (we did not ask for screenshots). Second, the study is of cross-sectional nature, hence it does not allow to infer causality from the variables investigated, we – for instance – can only assume that personality traits influence WhatsApp use, but such an assumption is based on theoretical thoughts and personality being a rather stable construct [[37], [38], [39]]. Although messengers can be seen as part of social media and the complete study was framed as a “WhatsApp study”, the four WtP questions did not explicitly mention WhatsApp in the question text, but social media in general. Also, this study focused on WhatsApp users, which might question the generalizability of the findings to other populations. On the other hand, recent research shows that WhatsApp represents an application that is being used by a majority of smartphone users across different generations in Germany [23]. A further limitation: The study investigated a scenario, which is a bit artificial, because at the moment users of WhatsApp cannot pay with money for a healthier service. Investigating the actual behavior, when such a choice would be available might come to different conclusions than the present research. It also needs to be mentioned that the questionnaires were administered in the German language and the data collection therefore largely took place in German-speaking countries. This means that the results cannot necessarily be applied to other study populations. In other countries the Willingness to Pay for social media and messengers may differ from the results presented here. Finally, we did not consider socio-economic variables such as income, which might be important to understand Willingness to Pay. Further, future studies should also take into account more theoretical considerations such as from uses and gratifications theory. Applications might differ in the gratifications they offer to their users and this might also impact upon Willingness to Pay. Addressing the mentioned limitations will help to further understand the overall generalizability of the here observed findings.

## 5 Conclusion

Social media was developed as a product by companies with the aim of making a profit. Over the past few decades, social media has become a worldwide phenomenon and a fundamental part of our society. The tech juggernauts operating behind social media have become the richest companies in human history by relying on surveillance capitalism with (un-)intended side effects for societies. Given the already mentioned problems arising from the current social media platforms operating with a data business model, it is highly relevant to think about solutions. Such solutions need to encompass that users can make self-determined decisions about their data, that users are not nudged towards longer use, and that social media does not undermine democracies. We believe it to be of particular importance to educate users about how the data business model works and how it impacts societies and individuals.

In the current work about 30% of the participants showed a Willingness to Pay (WtP), but about 70% were neutral or against payment models. The study showed that many factors have an influence on the WtP. Four of the five personality traits (Openness, Conscientiousness, Agreeableness, and Extraversion) correlated with WtP and/or the amount an individual is willing to pay per month for social media/messenger services. In addition to this, this study showed, that the formulation of questions and the described benefits from a payment model had an influence on the results. Further research is now needed to investigate in more detail whether there are other factors influencing the Willingness to Pay, how users can be sensitized to this topic and investigate other reasons for which users would want to pay for social media/messengers. The present findings might help nourish future discussions on alternative social media business models with empirical data.

## Data availability statement

This paper belongs to a larger project, which has been preregistered (although the present work was NOT preregistered): https://osf.io/pdvq5; the data can be found here: https://osf.io/j3kw9/.

## CRediT authorship contribution statement

**Christopher Kannen:** Writing – original draft, Methodology, Investigation, Formal analysis, Data curation, Conceptualization. **Cornelia Sindermann:** Writing – review & editing, Investigation. **Christian Montag:** Writing – review & editing, Investigation, Conceptualization.

## Declaration of competing interest

Dr. Montag reports no conflict of interest. However, for reasons of transparency Dr. Montag mentions that he has received (to 10.13039/501100008977Ulm University and earlier 10.13039/501100008131University of Bonn) grants from agencies such as the German Research Foundation (DFG). Dr. Montag has performed grant reviews for several agencies; has edited journal sections and articles; has given academic lectures in clinical or scientific venues or companies; and has generated books or book chapters for publishers of mental health texts. For some of these activities he received royalties, but never from gaming or social media companies. Dr. Montag mentions that he was part of a discussion circle (Digitalität und Verantwortung: https://about.fb.com/de/news/h/gespraechskreis-digitalitaet-und-verantwortung/) debating ethical questions linked to social media, digitalization and society/democracy at Facebook. In this context, he received no salary for his activities. Finally, he mentions that he currently functions as independent scientist on the scientific advisory board of the Nymphenburg group (Munich, Germany). This activity is financially compensated. Moreover, he is on the scientific advisory board of Applied Cognition (Redwood City, CA, USA), an activity which is also compensated. The other authors declare that they have no competing financial interests or personal relationships that could have appeared to influence the work reported in this paper.
