# Supplementary material for: On the Willingness to Pay for social media/messenger services taking into account personality and sent/received messages among WhatsApp users
Source: Heliyon. 2024 Mar 31;10(9):e28840. doi: 10.1016/j.heliyon.2024.e28840 (PMC11058879; doi:10.1016/j.heliyon.2024.e28840)
Supplement: Multimedia component 1 [file mmc1.docx]

**Comparison between calculations of Willingness to Pay groups (WtP groups)**

For the present study, we changed the way WtP groups were calculated slightly compared to the original study by Sindermann (2020). This current supplementary document explains the adjustments and reason why, and compares the results computed by the original and the adjusted grouping methods.

**WtP questionnaire**

The WtP questionnaire consists of four questions on a five-point Likert-scale (1. Strongly disagree, 2. Disagree, 3. Neutral, 4. Agree, 5. Strongly agree).

For further computations a WtP score is generated. This WtP score is the average of the answers to the four questions. With this score, each person can then be assigned to one of the following three WtP groups:

- Individuals that on average are not willing to pay.
- Individuals that on average stayed neutral.
- Individuals that on average are willing to pay.

The original method forms those groups as follows:

- Not Willing to Pay (1.00 <= WtP score <= 2.50); mathematical interval of [1, 2.5]
- Neutral (2.51 <= WtP score <= 3.50); mathematical interval of [2.51, 3.5]
- Willing to Pay (3.51 <= WtP score <= 5.00); mathematical interval of [3.51, 5.0]

(Sindermann et al., 2020)

This method followed the idea that cases with scores of 2.50 or 3.50, which could be rounded to be part of either the lower or higher group, should be put into the lower group. This is because this approach was seen as more conservative. In addition, this approach took into account previous literature showing that many individuals are not willing to pay. For instance, Sunstein (2018) reports that 15%-33% of individuals are not willing to pay for different social media platforms - as assessed by WTP - in a nationally representative US sample. Similarly, the WTP scores in the study by Sindermann et al. (2020) point toward a large share of individuals not being willing to pay for social media/messenger services. Consequently, the individuals with scores of 2.50 were seen as not willing to pay (instead of being neutral), and individuals with scores of 3.50 were seen as neutral (instead of willing to pay).

Without loss of information for this use case, the gaps of 0.01 between 2.50 and 2.51, and 3.50 and 3.51, can be removed when changing the intervals to:

- Not Willing to Pay (1.00 <= WtP score <= 2.50); mathematical interval of [1, 2.5]
- Neutral (2.50 < WtP score <= 3.50); mathematical interval of ]2.5, 3.5]
- Willing to Pay (3.50 < WtP score <= 5.00); mathematical interval of ]3.5, 5.0]

On a continuous scale, this would result in mathematically equal sections.

However, for the WtP score, the scale is discrete. Hence, taking the actual possible values into account, the sections are not exact the same:

The WtP score calculated from four questions is always a multiple of 0.25 and in the range between 1 to 5. Therefore, the Willing to Pay group has one possible value less that counts for that group (see Table 1, left side)

To balance the group sizes for not willing to pay and willing to pay better, we decided to include the value 3.5 to the Willing to Pay group. The mathematical description for this approach is as follows:

- Not Willing to Pay (1.00 <= WTP <= 2.50); mathematical interval of [1, 2.5]
- Neutral (2.50 < WTP < 3.50); mathematical interval of ]2.5, 3.5[
- Willing to Pay (3.50 <= WTP <= 5.00); mathematical interval of [3.5, 5.0]

As shown on the right side of Table 1, this leads from slightly unbalanced to now exactly balanced point ranges for the groups of willing and unwilling to pay with seven possible values per group.

Table 1: Possible points for Willingness to Pay score calculate from the four WtP questions and related group classification using the original and adjusted point ranges.

| **Original grouping method** | |  | **Adjusted grouping method** | |
| --- | --- | --- | --- | --- |
| Possible average points on WtP score | Group classification |  | Possible average points on WtP score | Group classification |
| 1.00 | Not willing to pay  (7 possible values) |  | 1.00 | Not willing to pay  (7 possible values) |
| 1.25 |  |  | 1.25 |  |
| 1.50 |  |  | 1.50 |  |
| 1.75 |  |  | 1.75 |  |
| 2.00 |  |  | 2.00 |  |
| 2.25 |  |  | 2.25 |  |
| 2.50 |  |  | 2.50 |  |
| 2.75 | Neutral  (4 possible values) |  | 2.75 | Neutral  (3 possible values) |
| 3.00 |  |  | 3.00 |  |
| 3.25 |  |  | 3.25 |  |
| 3.50 |  |  | 3.50 | Willing to pay  (7 possible values) |
| 3.75 | Willing to pay  (6 possible values) |  | 3.75 |  |
| 4.00 |  |  | 4.00 |  |
| 4.25 |  |  | 4.25 |  |
| 4.50 |  |  | 4.50 |  |
| 4.75 |  |  | 4.75 |  |
| 5.00 |  |  | 5.00 |  |

**Effects on results**

As shown in table 2, the descriptive statistics for WtP score and WtP groups are only slightly different.

For the group of individuals that are not willing to pay, the results do not change between both computation methods, because those groups were not affected by the changes.

Compared to the original approach, the new approach assigns more participants to the willing to pay group and less to the neutral group, as shown in table 3. For the results by Sindermann et al. (Sindermann et al., 2020) the percent numbers of individuals being categorized in the different groups change marginally with alternative cut-offs. 5.2 percentage points of the total participants are assigned to the group of individuals willing to pay for social media, instead of the neutral group, with the adjusted approach. For the data of the present work, the difference is a bit higher. 6.7 percentage points more of the total participants are assigned to the group of individuals willing to pay in this data set. In Figure 1. these results are illustrated graphically. For the interested reader, the data from Sindermann et al. (2020) and this paper are freely available, and different statistics with different cut-offs can be computed.

Table 2: Descriptive statistics of WtP scores and WtP groups, calculated using the original method and the new method with adjusted point ranges.

| **WtP score** | **N** | **Mean** | **Median** | **SD** |
| --- | --- | --- | --- | --- |
| **Sindermann et al. (2020)** | 210 | 2.65 | 2,75 | 1.13 |
| **Present work** | 2,924 | 2.72 | 2,75 | 1.12 |
| **WtP groups** | **N** | **Mean** | **Median** | **SD** |
| **Sindermann et al. (2020)**  **(original method)** | 210 | 0.28 | 0 | 0.80 |
| **Sindermann et al. (2020)**  **(adjusted method)** | 210 | 0.23 | 0 | 0.84 |
| **Present work**  **(original method)** | 2,924 | 0.24 | 0 | 0.81 |
| **Present work**  **(adjusted method)** | 2,924 | 0.17 | 0 | 0.86 |

Table 3: WtP groups computed by the original and new approach.

| **WtP groups** | **Not willing to pay** | **Neutral** | **Willing to pay** | **Difference** |
| --- | --- | --- | --- | --- |
| **Sindermann et al. (2020)**  **(original method)** | 104  (49.5%) | 61  (29.0%) | 45  (21.4%) | - |
| **Sindermann et al. (2020)**  **(adjusted method)** | 104  (49.5%) | 50  (23.8%) | 56  (26.7%) | 5.2pp |
| **Present work**  **(original method)** | 1383  (47.3%) | 860  (29.4%) | 681  (23.3%) | - |
| **Present work**  **(adjusted method)** | 1383  (47.3%) | 664  (22.7%) | 877  (30.0%) | 6.7pp |

Figure 1: Willingness to pay groups for the data sets of Sindermann et al. (2020) and the present work, computed with the original data point ranges by Sindermann et al. (2020) and the adjusted point ranges by the present work.


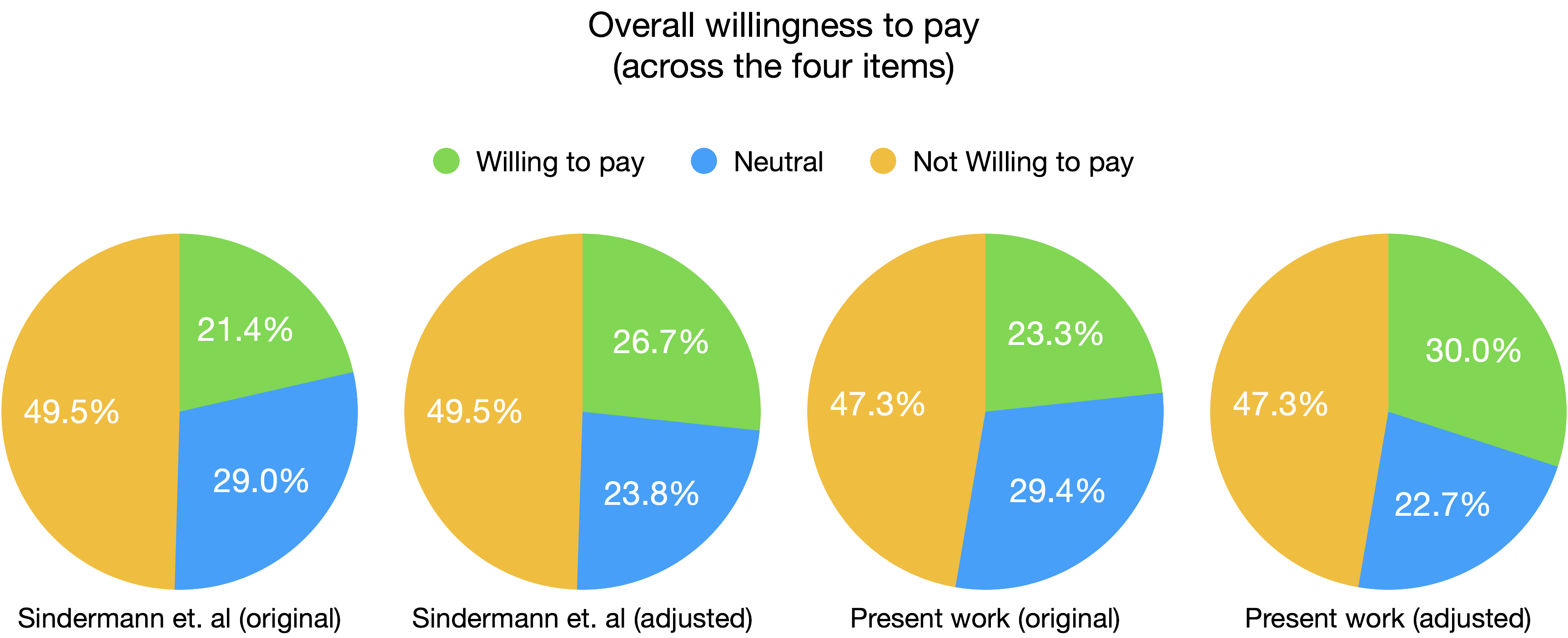


**Conclusion**

The adjusted approach for WtP grouping balances the groups for Not willing to pay and Willing to pay exactly. Although the previous method is also mathematical correct for continuous scales and the differences between both methods are rather low, we believe that the adjusted ranges, make the terms of and group classification in Willing to pay and Not willing to pay more comparable. Hence, we recommend using the adjusted intervals for the groups.

**References**

Sindermann, C., Kuss, D. J., Throuvala, M. A., Griffiths, M. D., & Montag, C. (2020). Should We Pay for Our Social Media/Messenger Applications? Preliminary Data on the Acceptance of an Alternative to the Current Prevailing Data Business Model. *Frontiers in Psychology*, *11:1415*(10.3389/fpsyg.2020.01415).

Sunstein, C. R. (2018). Valuing Facebook. *Behavioural Public Policy*, *4*(3), 370–381. https://doi.org/10.1017/bpp.2018.34
